# Monoamine oxidase B is elevated in Alzheimer disease neurons, is associated with γ-secretase and regulates neuronal amyloid β-peptide levels

**DOI:** 10.1186/s13195-017-0279-1

**Published:** 2017-08-01

**Authors:** Sophia Schedin-Weiss, Mitsuhiro Inoue, Lenka Hromadkova, Yasuhiro Teranishi, Natsuko Goto Yamamoto, Birgitta Wiehager, Nenad Bogdanovic, Bengt Winblad, Anna Sandebring-Matton, Susanne Frykman, Lars O. Tjernberg

**Affiliations:** 1 Karolinska Institutet, Department NVS, Center for Alzheimer Research, Division of Neurogeriatrics, Huddinge, Sweden; 2 Present address: Dainippon Sumitomo Pharma Co., Ltd, Drug Development Research Laboratories, Osaka, Japan; 3 National Institute of Mental Health, Klecany, Czech Republic; 4 Faculty of Science, Charles University in Prague, Prague, Czech Republic; 5 Department of Geriatric Medicine, University in Oslo, Memory Clinic, Oslo University Hospital, Oslo, Norway

**Keywords:** Alzheimer disease pathogenesis, Alzheimer disease, Amyloid β-peptide, Monoamine oxidase B, Intraneuronal amyloid β-peptide, γ-Secretase

## Abstract

**Background:**

Increased levels of the pathogenic amyloid β-peptide (Aβ), released from its precursor by the transmembrane protease γ-secretase, are found in Alzheimer disease (AD) brains. Interestingly, monoamine oxidase B (MAO-B) activity is also increased in AD brain, but its role in AD pathogenesis is not known. Recent neuroimaging studies have shown that the increased MAO-B expression in AD brain starts several years before the onset of the disease. Here, we show a potential connection between MAO-B, γ-secretase and Aβ in neurons.

**Methods:**

MAO-B immunohistochemistry was performed on postmortem human brain. Affinity purification of γ-secretase followed by mass spectrometry was used for unbiased identification of γ-secretase-associated proteins. The association of MAO-B with γ-secretase was studied by coimmunoprecipitation from brain homogenate, and by in-situ proximity ligation assay (PLA) in neurons as well as mouse and human brain sections. The effect of MAO-B on Aβ production and Notch processing in cell cultures was analyzed by siRNA silencing or overexpression experiments followed by ELISA, western blot or FRET analysis. Methodology for measuring relative intraneuronal MAO-B and Aβ42 levels in single cells was developed by combining immunocytochemistry and confocal microscopy with quantitative image analysis.

**Results:**

Immunohistochemistry revealed MAO-B staining in neurons in the frontal cortex, hippocampus CA1 and entorhinal cortex in postmortem human brain. Interestingly, the neuronal staining intensity was higher in AD brain than in control brain in these regions. Mass spectrometric data from affinity purified γ-secretase suggested that MAO-B is a γ-secretase-associated protein, which was confirmed by immunoprecipitation and PLA, and a neuronal location of the interaction was shown. Strikingly, intraneuronal Aβ42 levels correlated with MAO-B levels, and siRNA silencing of MAO-B resulted in significantly reduced levels of intraneuronal Aβ42. Furthermore, overexpression of MAO-B enhanced Aβ production.

**Conclusions:**

This study shows that MAO-B levels are increased not only in astrocytes but also in pyramidal neurons in AD brain. The study also suggests that MAO-B regulates Aβ production in neurons via γ-secretase and thereby provides a key to understanding the relationship between MAO-B and AD pathogenesis. Potentially, the γ-secretase/MAO-B association may be a target for reducing Aβ levels using protein–protein interaction breakers.

**Electronic supplementary material:**

The online version of this article (doi:10.1186/s13195-017-0279-1) contains supplementary material, which is available to authorized users.

## Background

Alzheimer disease (AD), the most common form of dementia, is an increasing global health problem as the number of affected cases increases with an aging population. The number of dementia cases over the world is estimated to be around 47 million in 2015 and is expected to reach 75 million by 2030 [1]. AD is a heterogeneous disease, and it is clear that amyloid β-peptide (Aβ) is a key molecule in the pathogenesis. Aβ is formed by proteolytic processing of the amyloid precursor protein (APP) by β-secretase followed by γ-secretase, which generates Aβ peptides of different lengths [2–4]. The longer Aβ peptides (Aβ42 and Aβ43) are more prone to aggregation and toxic than the shorter ones, such as Aβ40, which has been suggested to be protective [5]. The cores of amyloid plaques in the brains of AD patients are composed of fibrils formed by Aβ, mostly Aβ42 [6]. Early-onset familial AD (FAD) forms have an increased ratio of the longer Aβ species (Aβ42 or Aβ43) in relation to shorter ones (such as Aβ40) [7]. Pyramidal neurons in AD brains have higher levels of Aβ42 than neurons from control brains, and these intracellular levels of Aβ42 correlate with AD pathology [8, 9]. Positron emission tomography (PET) imaging using the Aβ amyloid ligand Pittsburgh compound B (PiB) show increased binding in AD brain before the onset of the disease. In addition, CSF levels of Aβ42 are decreased several years before the onset of AD [10]. With time, many other pathological changes appear, and it would be advantageous to start the AD treatment early. Current treatments include cholinesterase inhibitors (donepezil, rivastigmine and galantamine) and a glutamate antagonist (memantine). These drugs can delay some symptoms but not arrest the disease [1, 11] and there are currently no disease-modifying treatments for AD.

γ-Secretase is an important enzyme in AD due to its ability to liberate neurotoxic Aβ. However, targeting of γ-secretase with γ-secretase inhibitors has been found to lead to toxic side effects in clinical trials, probably due to the fact that γ-secretase can process around 100 substrates in addition to APP. One such substrate is Notch, whose cleavage by γ-secretase leads to the release of Notch intracellular domain (NICD), a physiologically important signaling molecule [12]. Consequently, treatment with γ-secretase inhibitors causes Notch-related side effects, including problems with the gastrointestinal system, skin and memory [13]. As an alternative to γ-secretase inhibition it would therefore be desirable to specifically reduce the production of neurotoxic Aβ species, without affecting Notch processing, by interfering with regulatory proteins. We used an unbiased method to identify γ-secretase-associated proteins that specifically upregulate Aβ production but not Notch processing. Thus it may be possible to pinpoint interactions for proteins that are associated with γ-secretase, which can be used as targets, and to identify or design interaction breakers as potential AD drugs.

Previous studies have shown that monoamine oxidase B (MAO-B) activity is increased in brain and platelets in AD [14, 15]. The correlation between this enzyme and AD has gained increased attention since recent PET studies showed enhanced binding of the MAO-B specific ligand ^11^C-deuterium-l-deprenyl (^11^C-DED) early in presymptomatic FAD cases, which was suggested to reflect enhanced astrocytosis [16]. However, the mechanism by which MAO-B affects AD pathogenesis is not known. Here, we report on the initial finding that MAO-B is associated with γ-secretase. Moreover, immunohistochemistry revealed that MAO-B is present in pyramidal neurons as well as glia cells in the frontal cortex and hippocampus in human brain, and that the levels of MAO-B are increased not only in astrocytes but also in neurons in AD. We used several different methods to verify that MAO-B is associated with γ-secretase in neurons, and regulates intraneuronal Aβ levels.

## Methods

### Purification, identification and characterization of γ-secretase-associated proteins

γ-Secretase was purified from synaptic membranes prepared from rat brain using γ-secretase inhibitor with a cleavable biotin group (GCB) and streptavidin beads, in the absence or presence of L-685,458 as a competing inhibitor, as described previously [17]. γ-Secretase-associated proteins that copurified with γ-secretase were identified by mass spectrometry, as described previously [17–19]. Briefly, the samples were subjected to tryptic digestion and the tryptic peptides were enriched and purified on C18 ZipTips (Millipore, Billerica, MA, USA). The purified tryptic peptides were analyzed by LC-MS/MS using an HPLC C18 chip system coupled to an electrospray ionization Ion Trap instrument (Agilent Technologies). MS spectra were collected between m/z 230 and 1800, and the five most intense ions in each scan were subjected to tandem mass spectrometry (MS/MS). Spectra were analyzed using Spectrum Mill Proteomics Workbench version A.03.03.078 (Agilent Technologies). The association of MAO-B with γ-secretase in human brain was also studied by coimmunoprecipitation (co-IP) with γ-secretase components, using anti-MAO-B as the precipitating antibody and anti-Nicastrin or anti-PS1 IgG as detecting antibodies, essentially as described previously [20]. Briefly, protein A-Dynabeads (Invitrogen) were incubated with microsomes from human brain cortex that had been preincubated with rabbit anti-MAO-B (M1821; Sigma-Aldrich) or another rabbit anti-MAO-B (OB1418, Dainippon Sumitomi Pharma (DSP), raised against the synthetic peptide NFWRTMDDMGREIPSDA corresponding to residues 117–133 of human MAO-B). The DSP antibody (OB1418) was validated by western blotting and immunocytochemistry (Additional file 1: Figure S1a–c). This antibody was also used to perform semiquantitative western blot (WB) analysis on postmortem brain homogenate prepared as described previously [20] from four AD cases with no investigated inheritance and four control cases. The M1821 antibody from Sigma-Aldrich is a commercially available antibody that has been validated previously (http://www.sigmaaldrich.com/catalog/product/sigma/m1821?lang=en&region=SE).

### Immunohistochemistry in thin sections from human brain

Immunohistochemistry was performed on the frontal cortex, hippocampus and entorhinal cortex from three control cases, two sporadic AD cases and one FAD case (Table 1). Brain blocks were fixed for 4 weeks in 4% buffered formaldehyde and embedded in paraffin. Samples (7 μm thick) were cut with a cryostat, autoclaved, deparaffinized and stained essentially as described previously [17], using a validated rabbit anti-MAO-B antibody (M1821; Sigma-Aldrich), followed by incubation with biotinylated anti-rabbit IgG and subsequently ABC-Elite HRP (Vector Laboratories). The stained samples were dehydrated and mounted in DPX (BDH Prolabo). Sections were counterstained with hematoxylin. For control samples the primary antibody was omitted.

**Table 1:** Control and AD postmortem cases used for immunohistochemistry

ID	Sex	Diagnosis	Heredity	Age at onset	Reached age	Braak stage	Known mutation
28	M	ND	na	na	78	na	na
84	M	ND	na	na	66	na	na
29	M	ND	na	na	86	na	na
11	M	FAD	Yes	47	68	V–VI	PSEN1
96	M	AD	No	55	64	V	na
40	F	AD	Yes	72	89	VI	na

*AD* Alzheimer disease, *F* female, *FAD* familial AD, *M* male, *na* not applicable, *ND* no detectable AD pathology

### Preparation of primary neurons and thin brain sections from mice

Primary hippocampal neurons were prepared from brains of E16.5 C57BL6 mice, seeded at a density of 7500 cells per well on the inner 10-mm microwell of poly-d-lysine-coated glass-bottom P35G-1.5-10-C culture dishes (MatTek Corporation) as described previously [21, 22] and were cultured for 14–21 days in vitro (DIV). Cortex cells used for small interfering RNA (siRNA) transfection experiments were prepared from the same embryonic mouse brains as the hippocampal cells and seeded in a similar manner but at a higher density of 10,000–15,000 cells per well and were cultured for 7 DIV. Both types of neurons were grown in selective Neurobasal medium containing 2% B27 (Invitrogen) and 1% l-glutamine (Invitrogen) at 37 °C in a cell incubator (humidified, 5% CO_2_). The cells were fixed in 10% neutral-buffered formalin (containing 4% formaldehyde) for 10 min at RT and stored in PBS at 4 °C. Immediately prior to immunocytochemistry and in-situ proximity ligation assay (PLA) experiments, the cells were permeabilized in 0.4% CHAPSO in PBS or neurobasal medium for 10 min at RT.

The brains of the female mice carrying the embryos used for culturing of hippocampal and cortex neurons were used for preparation of coronal sections (10 μm). These brains were fixed in 4% formaldehyde at 4 °C for >24 h and then placed stepwise in 10–20–30% sucrose/PBS for 1 day. The thin sections were prepared with cryostat and stored at –20 °C before use.

### Immunocytochemistry in mouse primary hippocampal neurons

The location of MAO-B in neurons was studied by immunocytochemistry and confocal microscopy. Fixed and permeabilized 21 DIV mouse primary hippocampal neurons were blocked with 10% normal goat serum (NGS) in PBS for 15 min at RT and incubated with rabbit anti MAO-B IgG (DSP) diluted 1:100, and in some cases mouse anti-NMDAr2B IgG2b (610416; BD Transduction Laboratories) diluted 1:100 in 5% NGS in PBS at 4 °C overnight. After washing two times each for 5 min, a secondary incubation step was conducted for 1 h at 37 °C in 5% NGS containing Alexa Fluor 488-conjugated mouse monoclonal anti-Tau-1 IgG diluted 1:100 (MAB 3420A4; Millipore), AbberiorSTAR635P-conjugated anti-rabbit IgG diluted 1:500 (2-0012-007-2; Abberior) and TRITC-conjugated Phalloidin diluted 1:200 (P1951; Sigma-Aldrich), or AbberiorSTAR635-conjugated anti-mouse IgG diluted 1:200 (2-0002-002-0; Abberior) and Alexa Fluor 488-conjugated anti-rabbit IgG (A11034; Life Technologies). Negative controls with only the secondary antibodies were also prepared. The final washing step was conducted three times each for 5 min in PBS containing 0.1% Tween-20 (PBST), three times each for 5 min in PBS and once for 1 min in water. The cells were mounted with ProLong gold antifade reagent (P36930; Life Technologies) and stored at 4 °C until imaging.

### PLA in neurons

PLA is a technique used to detect protein–protein associations with very high specificity and sensitivity in situ [23]. The proximity of two proteins is detected by utilizing probes (typically two different antibodies) conjugated to different oligonucleotide strands (where one strand is denoted PLUS and the other one is denoted MINUS) that can be ligated, amplified and labeled fluorescently. Because PLA detects protein–protein associations in situ, it avoids the risk of artifacts caused by overexpression of fluorescently tagged proteins used for instance in FRET experiments. It also avoids the risk for artifacts caused by homogenization procedures used for co-IP experiments. PLA was performed in mouse primary hippocampal neurons to determine whether γ-secretase is associated with MAO-B. γ-Secretase inhibitor with a transferrable biotin group (GTB), a previously described specific probe for labeling of mature γ-secretase [22, 24], was used. PLA was conducted essentially according to the manufacturer’s instructions (Olink Bioscience) and as described previously [22]. Briefly, 14 DIV fixed and permeabilized mouse primary hippocampal neurons were blocked with avidin and biotin according to the manufacturer’s instructions (Vector Laboratories) followed by blocking with the blocking solution (Olink Bioscience) for 30 min at 37 °C. The cells were incubated with 200 nM GTB diluted in antibody diluent (Olink Bioscience) for 1 h at 37 °C. GTB was photo-crosslinked by UV illumination, followed by washing five times each for 2 min with wash buffer A (Olink Bioscience). Next, the cells were incubated with PLA probes made in our laboratory from streptavidin and rabbit anti-MAO-B IgG (DSP) by Duolink probemaker kits minus and plus (Olink Bioscience), respectively, for 1 h at 37 °C, followed by washing five times each for 2 min with wash buffer A. For each experimental condition, we made two negative control sections, which were incubated with only one antibody or only GTB. All antibodies were diluted in the antibody diluent provided in the kit. Ligation and polymerization steps were performed according to the manufacturer’s instructions. To visualize neuronal structure, the cells were costained with Alexa Fluor 488-conjugated phalloidin (A12379; Life Technology) at 4 °C overnight prior to washing twice with PBS and mounting with DAPI-containing mounting medium from Olink Bioscience.

### PLA in mouse brain

Mouse brain sections prepared as already described were permeabilized for 10 min at RT with 0.4% CHAPSO. PLA was performed essentially as described earlier for neurons but with two principally different approaches. The first approach utilized unlabeled rabbit anti-MAO-B IgG (DSP) and mouse anti-PS1 loop IgG (Millipore), followed by PLA probes anti-mouse MINUS and anti-rabbit PLUS (Olink Bioscience), as described previously for neurons [25]. In the second approach, GTB and anti-MAO-B IgG (OB1418; DSP) were added in the first incubation step, followed by photo-crosslinking and washing as already described. In the second incubation step, PLA probe MINUS made in our laboratory by linking oligonucleotide MINUS probe to streptavidin and an anti-rabbit PLUS PLA probe (Olink Bioscience) were used. Ligation and polymerization steps were conducted as already described and according to the manufacturer’s instructions.

### PLA in human brain

PLA was performed on human brain in the same manner as for mouse brain (described earlier) using two complementary approaches, with the only difference being that for the GTB PLA the MAO-B antibody (OB1418; DSP) directly conjugated to an oligonucleotide was used (as shown in Fig. 4a). Prior to all antibody incubations, 10 μm thin sections of paraformaldehyde-fixed and paraffin-embedded human frontal cortex were deparaffinized, hydrated and further autoclaved in DIVA Decloaker (Histolab) at 120 °C for 20 min for epitope retrieval. After cooling, the sections were washed in tap water for 5 min. For the GTB sections, blocking with avidin and biotin (Vector Laboratories) was performed prior to the staining protocol, as described earlier for neurons. After the final washing step, sections were rinsed in PBS and incubated with Alexa Fluor 488-conjugated FluoroPan neuronal marker (Merck Millipore) and, for some slides, also with GFAP Alexa Fluor 594 conjugate (ThermoFisher Scientific) overnight at +4 °C. Slides were washed in PBS, dried in darkness and mounted with mounting media with DAPI.

### Gene silencing of MAO-B and measurements of Aβ and NICD in HEK cells

Silencer® Select Pre-Designed siRNA targeting MAO-B— ID s8493, sense siRNA GGACUUACACUCUUAGGAAtt and antisense siRNAUUCCUAAGAGUGUAAGUCCtg (MAO-B siRNA1); and ID s8494, sense siRNA GGACCAACCCAGAAUCGUAtt and antisense siRNA UACGAUUCUGGGUUGGUCCaa (MAO-B siRNA2)—were purchased from Ambion. siRNA treatments were performed essentially as described previously and the knock-down efficiency on mRNA levels were measured by real-time PCR (7500 Fast Real Time PCR system; Ambion) [19].

Secreted Aβ40 and Aβ42 levels were measured by a commercial sandwich ELISA (Immuno-Biological Laboratories, Gunma, and Wako Chemicals) in conditioned medium collected 24 h after siRNA transfection of HEK-293 cells overexpressing human wild-type APP 695 (HEK-APP cells), as described previously [17, 19].

The effects of MAO-B siRNA2 on Notch processing was studied on HEK-293 cells overexpressing the immediate substrate for γ-secretase, Notch ΔE (Notch ΔE cells), by western blotting followed by quantification of NICD, as described previously [17, 19]. The cell viability for the Aβ and NICD experiments were measured by Alamar Blue (Biosource Europe).

### Effects of overexpression of MAO-B in HepG2 cells on Aβ formation

HepG2 cells, which have a low endogenous expression of MAO-B and are easy to transduce, were cultured and transduced with BacMam viruses containing 20 multiplicity of infection (MOI) C99 (BacMam-C99), the immediate APP-derived substrate for γ-secretase, and 25, 50 and 100 MOI MAO-B (BacMam-MAO-B) as described previously [20]. Conditioned medium from the transduced cells was harvested after 24 h of incubation and the levels of Aβ40 were measured by a commercial FRET-based Aβ40 homogeneous time-resolved fluorescence (HTRF) assay (Cisbio Bioassays) [20]. After sampling of the culture supernatants for HTRF assay, the cell viability was analyzed by the WST8 assay (JM-K302; MBL) according to the manufacturer’s protocol. The relative cell viability was calculated by dividing the WST8 data for the test samples by those of the control BacMam-transduced HepG2 cells.

### Culturing and immunocytochemistry of BD3 and BD8 cells

Blastocyst-derived embryonic stem cells deficient for PS1 and PS2 (BD8 cells) and cells with one allele PS1 (BD3 cells) [26] were seeded at a density of 10,000 cells per well on poly-d-lysine-coated eight-well chamber slides (BD) in ES medium: Dulbecco’s modified Eagle’s medium (DMEM) supplemented with 10% (v/v) fetal calf serum, 1 mM sodium pyruvate, 0.1 mM β-mercaptoethanol and nonessential amino acids (Invitrogen). After culturing for 24 h the cells were fixed in 4% formaldehyde for 10 min at RT, permeabilized in 0.4% CHAPSO for 10 min at RT, blocked with 10% NGS in PBS for 15 min at RT and incubated with mouse anti-Aβ42 (clone G2-11, No. MABN12; Millipore), diluted 1:200 in 3% NGS in PBS, at 4 °C overnight. After washing five times each for 2 min, a secondary incubation step was conducted for 3 h at RT in 3% NGS containing AbberiorSTAR635-conjugated anti-mouse antibody (2-0012-007-2; Abberior) diluted 1:500. The stained cells were washed three times each for 5 min in PBS containing 0.1% TWEEN20 and three times each for 5 min with PBS, followed by rapid rinsing with MilliQ water prior to mounting with DAPI-containing mounting medium (Olink Bioscience) and covering with 24 × 50 mm cover glass No 1.5 (Marienfeld).

### Gene silencing in cortical neurons with siRNA

Silencing of the endogenous gene for MAO-B in 7 DIV mouse primary cortical neuronal cultures was performed with cationic lipid-mediated transfection. This 7 DIV stage of neurons was chosen because the transfection efficacy and cell survival were higher when the neurons were transfected at this stage than after culturing for a longer period. MAO-B siRNA2 (described earlier) and nontargeting sequence control siRNA (All Stars Negative siRNA; Qiagen) (control-siRNA) were diluted in nuclease-free water (Ambion) at 1 or 0.1 μM concentrations. Complexes of MAO-B siRNA2 or control-siRNA with Lipofectamine 3000 (Invitrogen) in Opti-MEM (10 μl of total volume) were formed at RT for 20 min. Cortical neurons were transfected directly into the inner microwells of the confocal plate by siRNA/Lipofectamine mixtures containing three different siRNA concentrations (0.36, 2.9 or 29 nM per 0.2 μl Lipofectamine 3000) in 70 μl Opti-MEM® (Gibco® by Life Technologies) solution and held at 37 °C in a cell incubator (humidified, 5% CO_2_). After 6 h incubation, 1.5 ml of medium (removed from the plates prior to transfection to minimize the transfection volume during the initial 6 h and kept at 37 °C) was added to each confocal plate, and neurons were incubated for 24 h under the same conditions. Cells were fixed with 4% formaldehyde for 10 min at RT, followed by washing with PBS, and were stored at 4 °C until PLA and staining were performed. To analyze the uptake/transfection efficacy of siRNA in the neurons, we performed control experiments using fluorescently labeled siRNA (All Stars Negative siRNA-AF488 (1027280; Qiagen) at the same concentrations and conditions as described earlier for the MAO-B-targeting siRNA.

### PLA and immunocytochemistry of siRNA-treated neurons

To visualize and quantify MAO-B in neurons by confocal microscopy after siRNA treatment, we chose PLA, which gives enhanced specificity and sensitivity and an amplified signal compared to traditional immunohistochemistry. By using PLA we were able to overcome difficulties caused by oversaturated nuclear area as well as difficulties to detect low MAO-B signals in small neurite projections. The quantitative MAO-B PLA was combined with traditional immunocytochemistry to quantify Aβ42. PLA is most known for detection of protein–protein interactions, but was used here for relative quantification of MAO-B levels using one single primary antibody to MAO-B followed by two secondary antibodies both directed to the same primary antibody but with two different oligonucleotides attached (one oligonucleotide MINUS and one oligonucleotide PLUS strand) [22, 25]. Briefly, the treated, fixed and permeabilized cells were blocked with blocking solution (Olink Bioscience) for 30 min at 37 °C. Incubation with primary antibodies, rabbit anti-MAO-B (DSP OB1418, 1:200) and mouse anti-amyloid β42 (clone G2-11, No. MABN12, 1:50; Millipore) was thereafter performed for 1 h at 37 °C. The second incubation step and the ligation and amplification reactions using Duolink™ II far red detection reagent (Olink Bioscience) were performed according to the manufacturer’s instructions. After the PLA protocol, TRITC-conjugated Phalloidin (P1951, 1:200; Sigma-Aldrich) and secondary anti-mouse IgG Alexa Fluor 488-conjugated antibody (A11001, 1:500; Invitrogen) were added followed by incubation overnight at 4 °C. Stained neurons were washed with PBS, mounted with DAPI-containing mounting medium (Olink Bioscience) and covered by 9 mm circular cover glass.

### Confocal visualization and analysis

Immunocytochemistry images were acquired in sequential mode on a Nikon A1RSi point scanning confocal inverted microscope using a 60× oil immersion objective with an image size of 1024 × 1024 pixels. Excitation lasers were 405, 488, 561 and 640 nm. The settings, including laser power intensities and detector gain, were chosen to optimize the dynamic range with no or limited fluorescence signal in the negative controls and no or limited saturation in the brightest samples.

All PLA samples and siRNA-treated samples were examined using a laser scanning confocal microscope (LSM 510 META; ZEISS) equipped with Zen 2009 software, using a plan-Neofluar 40×/1.3 oil immersion objective. For the relative quantification of neuronal MAO-B and Aβ levels in gene silencing studies, image acquisition was made with identical settings within the linear range of the detectors for cultures transfected with the same concentration of MAO-B siRNA and control-siRNA.

### Quantification of neuronal MAO-B and Aβ42 levels after siRNA silencing of MAO-B

Optical sectioning through the depth of the neurons was performed using Z-stacking. The transfection experiment was repeated twice, and at least five randomly chosen areas (224.8 μm × 224.8 μm) were captured in each sample. Image processing was performed with ImageJ 1.47 software (NIH). The regions of interest (ROIs) representing individual neurons were selected based on the phalloidin-stained areas, followed by binary segmentation using mean intensity auto threshold function. Subsequently, *z*-projections of average intensity images collected by Z-stack scanning at 0.54-μm intervals were used for quantification of MAO-B PLA signals and Aβ42 staining within individual ROIs. The corrected total cell fluorescence (CTCF) of MAO-B and Aβ42 signals was calculated according to the following equation:

CTCF = Integrated density – (area of selected cell × mean fluorescence of background readings).

Raw CTCF data were normalized on the basis of calculated CTCF levels from control-siRNA-treated samples (set as a value 1.0). CTCF data from cultures transfected with MAO-B-siRNA2 were normalized to CTCF values from the control-siRNA-treated samples (at corresponding siRNA concentrations). Student’s *t* test performed with GraphPad Prism software (San Diego, CA, USA) was used for analysis of differences in normalized CTCF levels in transfected neuronal cultures by MAO-B siRNA2 and relevant control-siRNA for both MAO-B and Aβ42 fluorescence signals. *p* < 0.01 was considered statistically significant. Data are presented as mean ± SEM.

## Results

### γ-Secretase is associated with MAO-B in synaptic membranes from brain

MAO-B was found to be a γ-secretase-associated protein by a method described previously for unbiased identification of γ-secretase-associated proteins [17–19]. Briefly, synaptic membranes from rat brain were solubilized in CHAPSO, incubated with the γ-secretase-binding probe GCB, followed by affinity purification using streptavidin beads and identification by LC-MS/MS. As a negative control, samples were incubated in the presence of a 50-fold molar excess of free L-685,458 inhibitor that competes with GCB binding to γ-secretase. In two out of three experiments in the samples without competing inhibitor, a database search of the MS/MS scan using the Spectrum Mill software identified a peptide sequence unique for MAO-B that was not found in any of the samples containing competing inhibitor. The peptide had an m/z of 813.42 (for a doubly-charged ion), corresponding to the amino acid sequence YVDLGGSYVGPTQNR (Additional file 1: Figure S1d). The association of MAO-B with γ-secretase was confirmed by co-IP, showing that nicastrin and the C-terminal fragment of Presenilin 1 (PS1-CTF) coimmunoprecipitated with MAO-B in microsomes prepared from postmortem human cortex (Additional file 1: Figure S1e). MAO-B levels in homogenates from the frontal cortex of postmortem control and AD brain, evaluated by semiquantitative WB analysis, showed increased levels in the AD cases (Additional file 1: Figure S2).

### MAO-B is expressed in neurons in human brain and is increased in AD

**Fig. 1:**
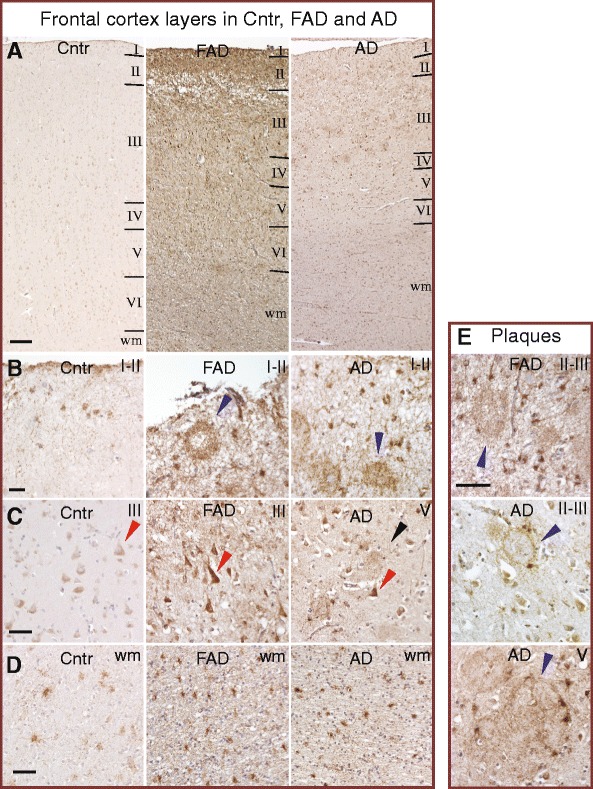
MAO-B immunohistochemical staining of thin sections from the frontal cortex of Control (*Cntr*), FAD and AD brains. **a** Typical images of the different layers of frontal cortex from one each of a Cntr, FAD and AD case are shown in images taken at 4× magnification. *Scale bar*, 200 μm. **b** Layers I–II with plaque staining (*blue arrows*) in the FAD and AD cases, imaged at 20× magnification. *Scale bar*, 50 μm. **c** Layer III with pyramidal neurons that are intensively stained in FAD and AD (20× magnification). Examples of pyramidal neurons shown by *red arrows*. Note the neurite staining (*black arrow*). *Scale bar*, 50 μm. **d** In the white matter (*wm*) the density of stained astrocyte-like cells is more intensive in FAD and AD (20× magnification). *Scale bar*, 50 μm. **e** Plaques in layers II–III and V surrounded by MAO-B positive cells in FAD and AD (40× magnification). *Blue arrows* point at plaques. The white balance for the background was adjusted in Photoshop, in a similar manner for all raw images, using a color sample tool and color balance to adjust highlights. *Scale bar*, 50 μm. *AD* Alzheimer disease, *FAD* familial AD (Color figure online)

To find out in which cells MAO-B is expressed and whether there are differences between AD and control brains, thin sections from the frontal cortex, hippocampal CA1 and entorhinal cortex were subjected to immunohistochemistry using a validated antibody against MAO-B combined with hematoxylin counterstaining. A representative overview of the staining of MAO-B in frontal cortex layers I–VI and white matter is shown from one control, one AD case and one FAD case (Fig. 1a). Similar observations were made in the other control and AD cases presented in Table 1. Atrophy of the cortex was clearly observed in the AD and FAD cases, with thinner layers I–IV compared to the control. Cellular MAO-B staining was more intense in AD and FAD cases compared to the control throughout the cortex region. Staining of plaques was observed in layers I–II of the FAD and AD cases (Fig. 1b). A magnified image of the pyramidal neurons in layer III showed staining in the soma and dendrites, which was considerably more intense in the FAD and AD cases than in the controls (Fig. 1c). Increased staining of astrocytes and microglia were observed in white matter from AD and FAD cases (Fig. 1d) and MAO-B positive cells surrounding plaques were observed in both FAD and AD cases (Fig. 1e). Most of those cells have a morphology that resembles astrocytes.

**Fig. 2:**
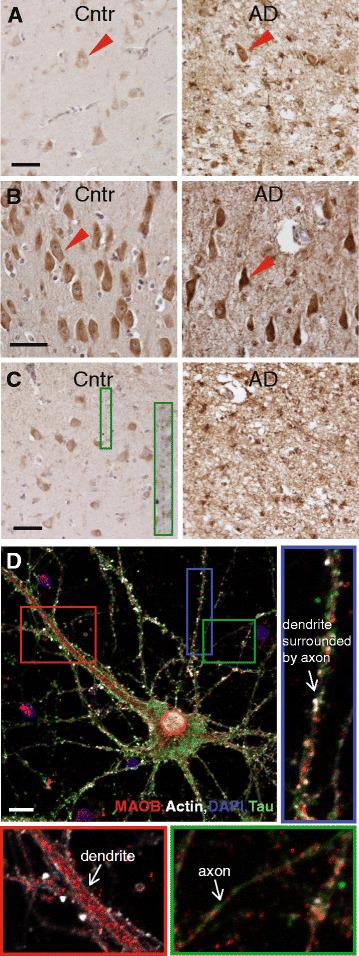
MAO-B immunohistochemistry in thin sections from hippocampus CA1 and entorhinal cortex regions of Cntr and AD brains. **a** Hippocampus CA1 images taken at 20× magnification are shown from a Cntr case (*left panel*) and an AD case (*right panel*). Note a neuronal loss in AD and intensive staining in the remaining neurons, including fibers, and astrocyte-like cells. Examples of pyramidal neurons are marked by *red arrows. Scale bar*, 50 μm. **b** Images of CA1 regions with many pyramidal neurons taken at 40× magnification in a Cntr (*left panel*) and AD (*right panel*) brain. Note a bigger variability in the pyramidal neuron staining of AD case while neurons in the control case are more evenly stained. *Red arrows* point at examples of pyramidal neurons. *Scale bar*, 50 μm. **c** Entorhinal cortex, layer II image taken at 20× magnification from a Cntr (*left panel*) and AD (*right panel*) brain. Zoomed inset in the left panel shows axonal staining. Note an island-like neuronal staining in the control case that is diminished and replaced by astrocyte-like staining due to the neuronal loss and advanced neurodegeneration in AD. *Scale bar*, 50 μm. **d** Immunocytochemistry in mouse primary hippocampal neurons shows axonal, dendritic and somal locations of MAO-B. The neurons were stained for Phalloidin to visualize F-actin and the neuronal structure (*white*), DAPI to visualize nuclei (*blue*), Tau-1 to visualize axons (*green*) and MAO-B (*red*). *Red zoomed inset* shows MAO-B staining in a thick (apical) dendrite. *Blue zoomed inset* shows MAO-B staining in a narrower dendrite. *Green zoomed inset* shows MAO-B staining in an axon. Images were taken with a Nikon A1RSi point scanning confocal with a 60× oil immersion objective. *Scale bar*, 10 μm. The axonal image is the sum of a *z*-stack whereas all other images are single planes. *AD* Alzheimer disease (Color figure online)

In hippocampal CA1, neurons and glia cells were MAO-B positive and more intensively stained in AD than in the controls (Fig. 2a). There was a loss of neurons in the AD cases, and the remaining degenerating neurons in CA1 in AD had intensive MAO-B staining (Fig. 2b). MAO-B staining was observed in the soma, dendrites and axons. The entorhinal cortex also showed a more intensive staining in glia cells and neurons in AD than in the control (Fig. 2c), and a large loss of neurons were observed in many areas of the AD cases.

### MAO-B is present in soma, dendrites and axons in primary hippocampal neurons

To study the neuronal distribution of MAO-B in further detail, we performed immunocytochemistry in primary hippocampal neurons. In addition to anti-MAO-B IgG, cells were stained with anti-tau-1 IgG as a marker for axons, DAPI as a marker for nuclei and phalloidin, which binds to fibrillary actin (F-actin). MAO-B was present in soma, dendrites and axons (Fig. 2d). As described previously, mouse primary hippocampal neurons contain axons that are directed along with (in parallel and around) dendrites as well as between dendrites [24], and here we found that MAO-B was present in both of these.

**Fig. 3:**
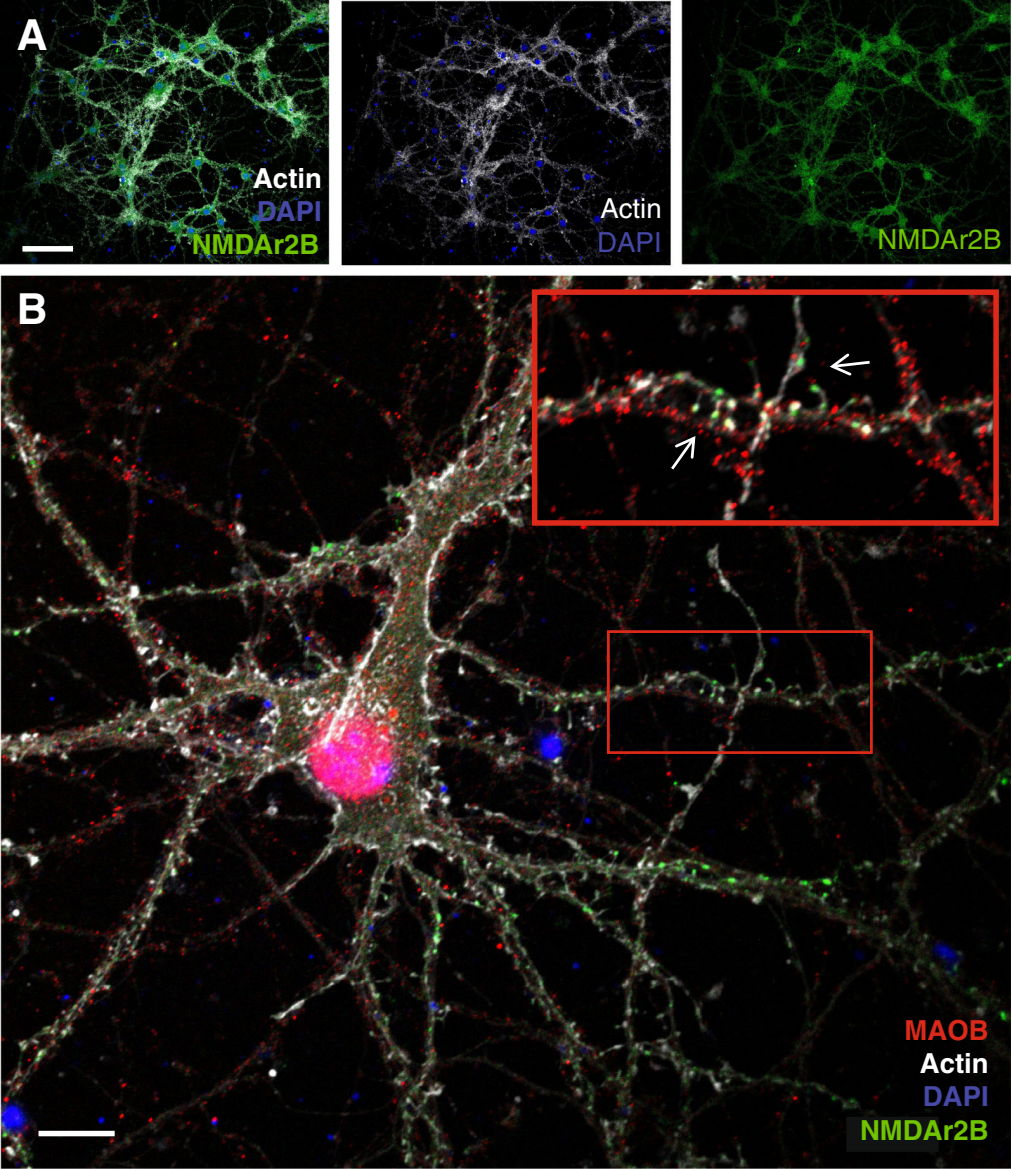
Immunocytochemistry in primary neurons to elucidate the MAO-B location in relation to the synapse. Primary hippocampal neurons were stained with Phalloidin to visualize fibrillar actin and the neuronal structure (*white*), DAPI to visualize nuclei (*blue*), anti-NMDAr2B to visualize the postsynaptic side of glutamatergic synapses (*green*) and anti-MAO-B (*red*). **a** Low-magnification overview imaged with a 20× objective, to visualize that most or all of the pyramidal neurons in our cell cultures contain NMDAr2B. **b** High-magnification image taken with a 60× oil immersion objective. *Inset* shows a zoomed part of the large image that was enriched with a high density of NMDAr-containing dendritic spines and MAO-B staining on the opposing axons. The intensity of the *red channel* in the *inset* was enhanced using image J (Color figure online)

To find out whether MAO-B was localized close to synapses, we also stained neurons with antibodies for MAO-B and NMDAr2B, together with DAPI and phalloidin. NMDAr2B is the glutamate-binding subunit of the NMDAr, and is to a large extent present in the postsynaptic plasma membranes of glutamatergic synapses [27]. Most, possibly all, of the pyramidal neurons in our cellular cultures contained NMDAr2B (Fig. 3a). By high magnification confocal microscopy studies, using a 60× oil immersion objective and optimal zooming, we observed an enrichment of NMDAr2B at many of the dendritic spines. Interestingly, a high extent of MAO-B staining was observed at axons in close proximity to NMDAr2B-containing spines (Fig. 3b).

### γ-Secretase is associated with MAO-B in primary neuronal cultures

**Fig. 4:**
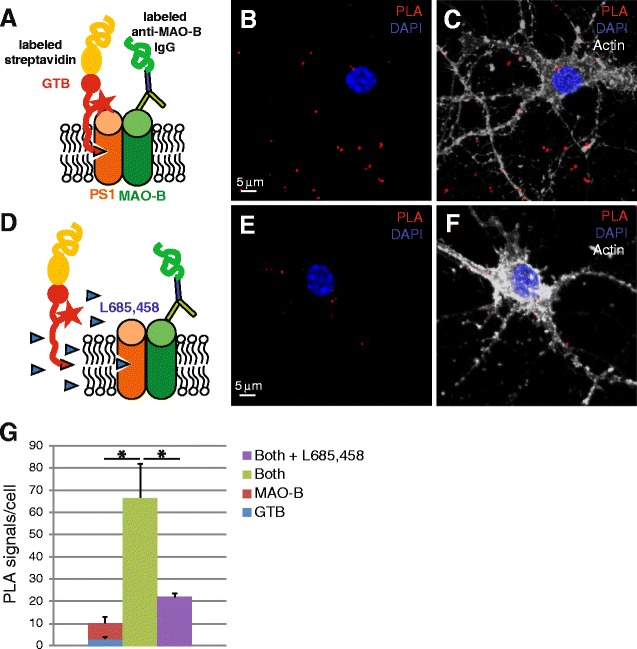
In-situ proximity ligation assay (*PLA*) in cultured neurons showing association between MAO-B and active γ-secretase. **a** Schematic drawing of the PLA used to study the association between active γ-secretase and MAO-B using GTB as a PLA probe. **b** Typical confocal image showing the PLA signals (*red dots*) representing the association between active γ-secretase and MAO-B and DAPI staining to visualize nuclei (*blue*). *Scale bar*, 5 μm for all four images. **c** Same image as in **b** but with phalloidin stain (*gray*) to visualize also the cell structure. **d** Schematic of the setup for competition of the PLA signal in the presence of excessive amounts of the free γ-secretase inhibitor L-685,458. A typical image of the PLA signals in the presence of competitor is shown without (**e**) and with (**f**) phalloidin stain. Quantification of the PLA signals was done using the Duolink image tool (**g**). Mean values ± SEM from quantifications of at least three cells from three different experiments are shown. Statistical significance was calculated with one-tailed, two-sample unequal variance *t* test. *p < 0.05 (Color figure online)

Because immunohistochemistry and immunocytochemistry studies showed MAO-B staining in pyramidal neurons, and the staining intensity was increased in AD as compared to control cases, we chose to use primary neurons for detailed interaction studies with PLA. In our previous study comparing the interaction of γ-secretase with several γ-secretase-associated proteins, antibody-based PLA indicated that MAO-B is associated with PS1 in primary neurons [25]. In this study we investigated the interaction using the γ-secretase probe GTB and an oligonucleotide-conjugated MAO-B antibody for PLA. The experimental setup, displayed in Fig. 4a, allows competition experiments using excessive amounts of free inhibitor as an additional negative control (Fig. 4d). Because GTB binds to the active site of γ-secretase, only the fully assembled γ-secretase complex, not the individual components, is detected with this probe [22]. Furthermore, the direct conjugation of the antibody lowers the maximum distance between two molecules for which an interaction can be observed to around 20 nm. PLA signals were observed (Fig. 4b, c) that were competed for by the free inhibitor (Fig. 4e, f). The number of PLA signals were 6.5-fold higher for the interaction sample than the sum of the negative controls (Fig. 4g), demonstrating that MAO-B is associated with mature γ-secretase containing an active site.

### γ-Secretase is associated with MAO-B in mouse brain

**Fig. 5:**
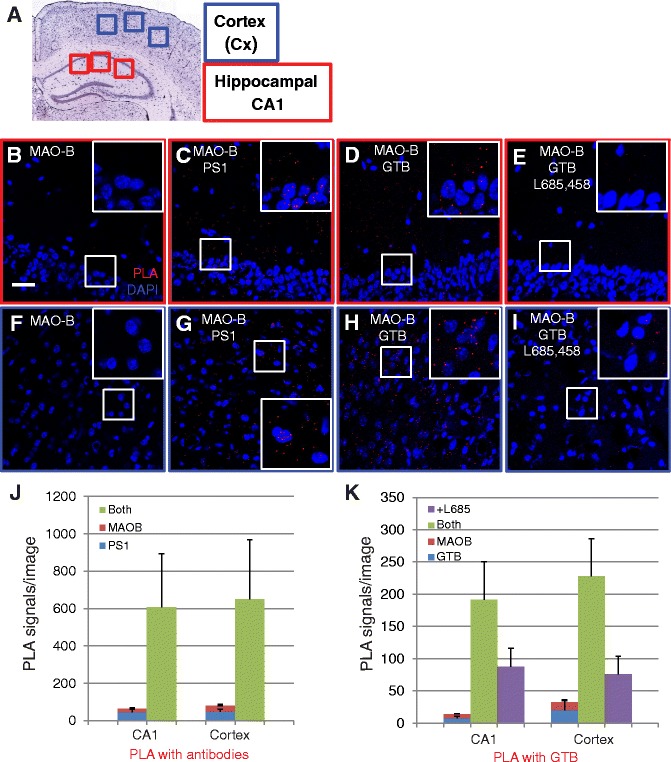
Proximity ligation assay (*PLA*) to visualize the PS1/MAO-B and γ-secretase/MAO-B interactions in mouse brain. Female adult mouse brains were fixed in formalin, and 10-μm sections were cut using a cryostat and stored at −20 °C prior to PLA assay. **a** Cross-section of a mouse brain showing the regions chosen for PLA imaging: cortex (*blue squares*) and hippocampal CA1 (*red squares*). **b**–**e** PLA in mouse hippocampal CA1 to visualize the association between PS1 and MAO-B (using a PS1 and an MAO-B antibody) and between active γ-secretase and MAO-B (using GTB and an MAO-B antibody) as well as negative controls (only MAO-B antibody and competition in the presence of L-685,458). **f**–**i** PLA in mouse cortex (with the same antibody and GTB combinations as in **b–e**). *Insets* are enlarged parts of the original images to clearly visualize the PLA signals (note that the small size of PLA signals makes it difficult to see them on printouts). **j** Quantification of the PLA signals in hippocampal CA1 and cortex for the PS1/MAO-B association. **k** Quantification of the PLA signals in hippocampal CA1 and cortex for the association between mature γ-secretase and MAO-B. Quantification of the number of PLA signals was done using the Duolink image tool. Mean values ± SE from at least three images in 10 experiments (traditional PLA) or three experiments (GTP-PLA) are shown (Color figure online)

To study the distribution of the γ-secretase/MAO-B association in different brain regions, we used thin sections from cortex and hippocampal CA1 regions from mouse brain (Fig. 5a). Negative controls, using only anti-MAO-B IgG in the hippocampus (Fig. 5b) and cortex (Fig. 5f) regions, gave, as expected, only few signals. PLA showed an association both in the hippocampus (Fig. 5c) and the cortex (Fig. 5g) when the γ-secretase/MAO-B interaction was detected with antibodies directed to MAO-B and PS1. In addition, PLA showed an association both in the hippocampus (Fig. 5d) and cortex (Fig. 5h) when the active site probe GTB and an MAO-B antibody were used to detect the interaction. With the latter approach, most of the PLA signals could be competed for by an excess of the free inhibitor L-685,458 (Fig. 5e, i). The PLA dots were quantified from at least three images in 10 or three experiments for traditional PLA and GTB-PLA, respectively (Fig. 5j, k). GTB-PLA gave at least as many signals (14-fold and 7-fold higher for the interaction sample than the sum of the negative controls in CA1 and cortex, respectively) as traditional PLA (10-fold and 8-fold higher for the interaction sample than the sum of the negative controls in CA1 and cortex, respectively). These data indicate that the majority of MAO-B association occurs with mature γ-secretase (and not immature or free PS1) in the mature mouse brain.

### MAO-B is associated with γ-secretase in human frontal cortex sections

**Fig. 6:**
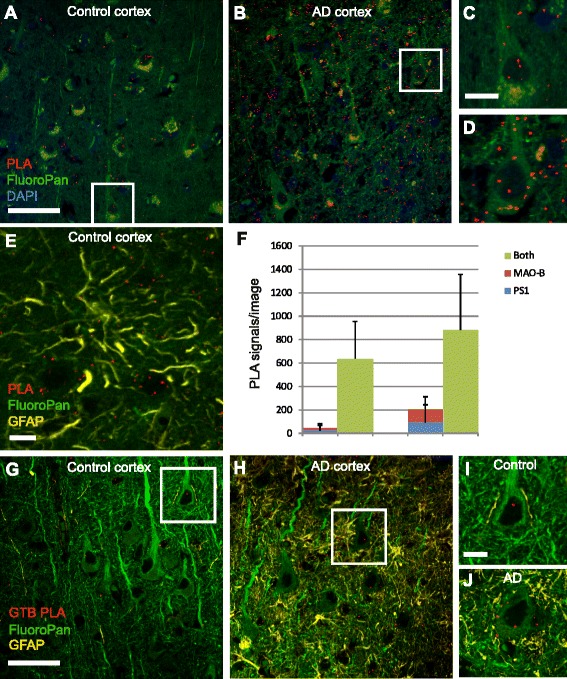
Proximity ligation assay (*PLA*) to visualize the PS1/MAO-B and γ-secretase/MAO-B interactions in postmortem human brain cortex. PLA using the traditional setup, with antibodies for MAO-B and PSI, in neurons in Cntr (**a**, **c**) and AD (**b**, **d**). PLA signals are shown in *red*. The samples were costained with DAPI to visualize nuclei (*blue*) and FluoroPan marker to visualize the neuronal structure (*green*). An image of a Cntr sample showing both neuronal stain (*green*) and astrocytic stain (*yellow*) shows that a few PLA signals were also present in astrocytes (**e**). PLA signals/image from the standard PLA setup were quantified using at least three images from four different experiments using the Duolink image tool (**f**).The human cortex was also subjected to PLA using the GTB-PLA method and directly labeled MAO-B antibody to visualize the association between active γ-secretase and MAO-B in control brain (**g**, **i**) and AD brain (**h**, **j**) where neurons are visualized with FluoroPan neuronal marker (*green*) and astrocytes with GFAP (*yellow*). *White scale bar*, 50 μm (**a, b, g, h**) and 10 μm (**c**, **d**, **e, i, j**). *AD* Alzheimer disease (Color figure online)

Next, we used PLA to study whether we could observe the interaction between MAO-B and γ-secretase in thin sections of human frontal cortex from control and AD cases. We utilized both traditional PLA using MAO-B and PS1 antibodies as well as GTB-PLA employing directly conjugated MAO-B antibody and the active-site probe GTB combined with DNA-conjugated streptavidin for detection of mature γ-secretase. We detected PLA signals in the brain sections probed with PS-1 and MAO-B antibodies in control brain (Fig. 6a, c) and in AD brain (Fig. 6b, d). The signals were detected in neurons (Fig. 6a–d) and to some extent in astrocytes (Fig. 6e), as visualized by using the astrocytic marker GFAP (Fig. 6e). The detected PLA signal was several-fold higher in the sections incubated with both PS1 and MAO-B antibodies as compared to the negative controls (sections incubated with only one antibody) (Fig. 6f). There was a high extent of variation in the PLA signals in human brain. Therefore we were unable to detect any significant differences in the amount of PLA signals between control brain and AD brain. As for the standard PLA, GTB-PLA performed on frontal cortex sections generated PLA signals in neurons and also to some extent in astrocytes in control brain (Fig. 6g, i) and AD brain (Fig. 6h, j). Staining with the FluoroPan neuronal marker and the astrocytic marker GFAP showed a loss of healthy neurons and an increase in the number of reactive astrocytes in AD compared to control brain, as expected (Fig. 6g–j).

### Effects of siRNA interference of MAO-B on Aβ production and Notch processing

**Fig. 7:**
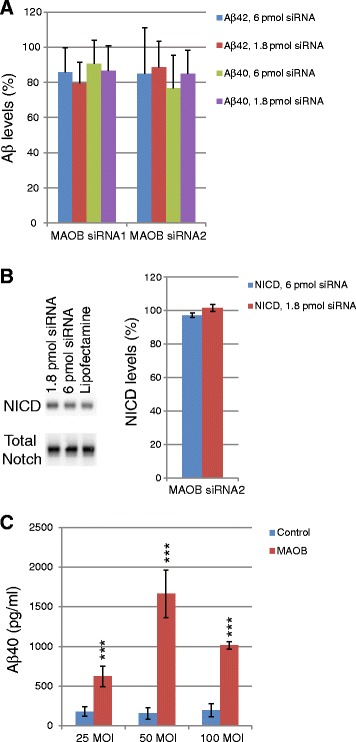
Effect of silencing and overexpression of MAO-B on Aβ production and Notch processing in HEK293 cells and HEPG2 cells. **a** HEK-APP cells were transfected by lipofection with two different siRNAs targeting MAO-B and after 24 h the levels of Aβ40 and Aβ42 in the conditioned medium were measured by ELISA. **b** HEK cells overexpressing Notch lacking the ectodomain (Notch ΔE) were transfected with siRNA targeting MAO-B and after 3 days of transfection the cells were lysed, subjected to WB analysis and the NICD levels were determined from CCD camera images of the WB. A typical WB is shown, *n* = 3. **c** Effect of overexpression of MAO-B and C99 in HEPG2 cells on Aβ production. HepG2 cells were coinfected with BacMam-C99 and BacMam-MAO-B. Levels of Aβ40 secreted into the conditioned medium were measured by a HTRF assay. Data shown represent mean values ± SD (*n* = 8). Significance was determined using two-tailed, two-sample unequal variance *t* test. ***p < 0.001. *Aβ* amyloid β-peptide, *MOI* multiplicity of infection, *NICD* Notch intracellular domain, *siRNA* small interfering RNA

Because MAO-B was found to be associated with γ-secretase, we investigated whether MAO-B has any effect on Aβ production or Notch processing. First, we silenced the expression of MAO-B in HEK cells and studied the effect on secreted Aβ (by ELISA) and NICD formation (by western blot). HEK cells overexpressing human wild-type APP (HEK-APP cells) were used for Aβ measurements, whereas HEK-293 cells overexpressing the immediate substrate for γ-secretase (Notch ΔE cells) were used for NICD measurements. Two different MAO-B siRNAs were used for the experiments. Knock-down efficacies for both siRNAs were 93% at 1.8 pmol and 95–96% at 6 pmol siRNA. Viability, determined by Alamar Blue, was >88% of the lipofectamine control for all experiments included (*n* = 3–4), and the data were corrected for Alamar Blue data. Small reductions were observed in the amount of secreted Aβ40 as well as Aβ42 (Fig. 7a). These reductions were nonsignificant when analyzing the effects of different siRNAs individually by Student’s *t* test, but all showed a similar trend; that is, a decrease in the levels of secreted Aβ40 and Aβ42. In contrast, no reduction in Notch processing was observed using MAO-B siRNA2 (Fig. 7b).

### Overexpression of MAO-B enhances secreted Aβ levels

To investigate whether increased expression of MAO-B could lead to enhanced Aβ production, we measured secreted Aβ after overexpression of both MAO-B and the immediate γ-secretase substrate C99 (the BACE-1 cleavage product of APP) in HepG2 cells. This is a liver-derived cell line that has a low endogenous expression of these proteins, and is suitable for transduction by being tolerable and giving good transduction efficacy. The cells were coinfected with BacMam-C99 and BacMam-MAO-B. Cell viability measured by WST8 showed that the viability was unchanged by the treatment. The overexpression of MAO-B was confirmed by western blot analysis (Additional file 1: Figure S1f). Secreted Aβ40 in the culture supernatants was measured by the HTRF assay (which is available for Aβ40 but not Aβ42) [20]. Interestingly, increased MAO-B expression resulted in significantly increased Aβ40 generation in the presence of the expressed immediate substrate C99 at all DNA doses used (Fig. 7c).

### siRNA silencing of MAO-B reduces intracellular Aβ42 levels in primary neurons

**Fig. 8:**
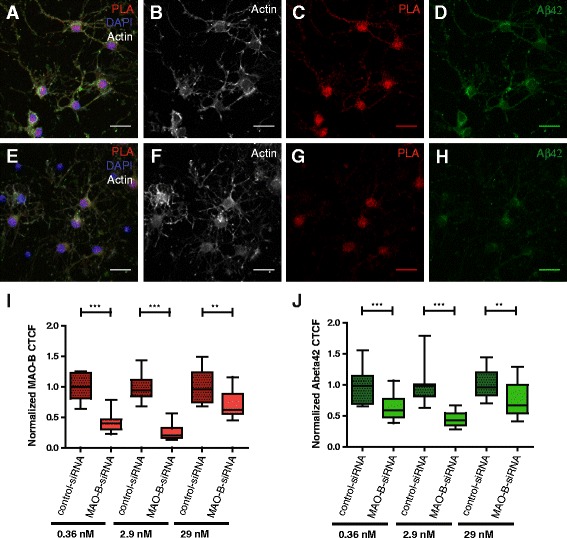
MAO-B silencing by siRNA transfection and its effect on intracellular Aβ42. The effect was investigated for three concentrations of control-siRNA/MAO-B siRNA2 (0.36, 2.9 and 29 nM). Localization of MAO-B staining by proximity ligation assay (*PLA*; *red*) in neurons, intracellular Aβ42 staining (*green*), DAPI nuclear staining (*blue*) and F-actin-binding TRITC-conjugated phalloidin (*gray*) is illustrated for both control-siRNA-treated (**a**) and MAO-B siRNA2-treated (**e**) cells. TRITC-conjugated phalloidin staining shows no significant alterations in neuronal structures after siRNA lipofection for both control-siRNA-treated (**b**) and MAO-B siRNA2-treated (**f**) cells. Representative images of MAO-B suppression by 2.9 nM MAO-B siRNA2 and the effect on intracellular Aβ42 levels in 8 DIV neurons (**g**, **h**) compared with the same concentration of control-siRNA-treated samples (**c**, **d**). MAO-B and Aβ42 fluorescence signals, quantified as normalized corrected total cell fluorescence (*CTCF*, see Methods), are shown in box plot graphs. Data from single cells in two different experiments is shown in Additional file 1: Figure S4. Normalized data are presented as mean values ± SEM. Significance was determined using two-tailed, two-sample unequal variance *t* test for the same concentrations of control-siRNA (normalized as value 1.0) and MAO-B siRNA2. The decreased protein level of MAO-B (*red*) in MAO-B siRNA transfected neurons compared to control-siRNA transfected neurons is shown (**i**). The effect of MAO-B silencing on intracellular Aβ42 levels (*green*) by MAO-B siRNA2 silencing compared with a control-siRNA-treated sample is illustrated in (**j**). Z-projections of maximum intensity images collected by Z-stack scanning at 0.54-μm intervals with a laser scanning confocal microscope (LSM 510 META; ZEISS) are shown, *scale bar* = 30 μm. **p < 0.01; ***p < 0.001. *siRNA* small interfering RNA

**Fig. 9:**
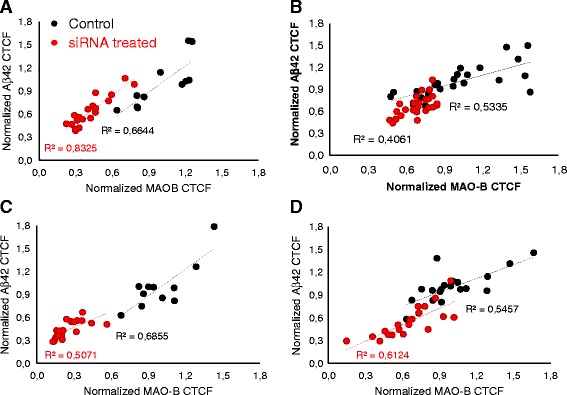
Correlation of Aβ42 and MAO-B cellular fluorescence in cortex neurons. Correlation plots of normalized MAO-B and Aβ42 CTCFs data of individual cells from MAO-B siRNA2-treated (*red circles*) and control- siRNA-treated (*black circles*) cultures from both transfection experiment I (**a**, **c**) and transfection experiment II (**b**, **d**). **a, b** 0.36 nM siRNA; **c, d** 2.9 nM siRNA. Coefficients of determination (*R* ^2^) data for each treatment group were calculated. *Aβ* amyloid β-peptide, *CTCF* corrected total cell fluorescence

The small reduction of secreted Aβ observed in MAO-B silencing studies with HEK-APP cells could be caused by a low extent of PS1/MAO-B association in this cell type [25]. In contrast, a high degree of PS1/MAO-B association was observed in cultured neurons. Because intraneuronal Aβ42 is increased in AD and believed to be neurotoxic [9] we investigated whether the intraneuronal Aβ42 levels in primary neurons can be significantly affected by MAO-B silencing, and we used MAO-B siRNA2 to silence MAO-B expression in primary cortex neurons. Quantification was done on a single cell basis, allowing quantification of intracellular MAO-B, as well as Aβ42, fluorescence intensity in individual cells. The specificity of the MAO-B antibody was confirmed as already described (Additional file 1: Figure S1) and the specificity of Aβ42 staining was confirmed by immunocytochemistry in BD8 cells lacking PS1 and PS2, compared with BD3 cells containing one PS1 allele (Additional file 1: Figure S3).The cellular morphology was unchanged by the treatment (Fig. 8a, b, e, f), while MAO-B protein expression levels were successfully reduced upon siRNA silencing (Fig. 8c, g). The effect of MAO-B protein levels on intracellular Aβ42 (Fig. 8d, h) was evaluated in a single-cell analysis, showing a significant reduction of Aβ42 levels in transfected neurons with reduced MAO-B protein expression (Fig 8i, j; Additional file 1: Figure S4). Three different concentrations of MAO-B siRNA2 and control-siRNA were used in two different transfection experiments. Interestingly, we observed lower or no effect on both MAO-B and Aβ42 levels at the highest siRNA concentration (29 nM) (Fig. 8i, j; Additional file 1: Figure S4). In analogy, using AlexaFluor 488-labeled negative control-siRNA, we observed fluorescence in most of the cells, showing that the majority of the cells took up siRNA. However, accumulation in vesicular structures was observed at the highest siRNA concentrations used (Additional file 1: Figure S5). Notably, single cell quantitative analysis revealed that MAO-B protein and intracellular Aβ42 peptide levels correlate not only in transfected but also in nontransfected cells (Fig. 9), suggesting that endogenous MAO-B regulates Aβ production.

## Discussion

Our finding that MAO-B is associated with γ-secretase and affects Aβ production provides a molecular link to the previously reported connection between MAO-B and AD pathogenesis. Here, immunohistochemistry showed staining of MAO-B in neurons as well as glia cells in the frontal cortex, hippocampus and entorhinal cortex, and an increase in the staining intensity was observed both in sporadic and FAD cases as compared to controls. The enhanced MAO-B staining in astrocytes and the presence of such astrocytes surrounding plaques in AD is in agreement with previous studies [28, 29]. In contrast, our findings that MAO-B levels increase in pyramidal neurons in the frontal cortex, hippocampus CA1 and entorhinal cortex in AD have to our knowledge not been reported previously.

The discovery that MAO-B is a γ-secretase-associated protein in the brain, first made by affinity pull-down using a γ-secretase-specific probe, and subsequently confirmed by co-IP of γ-secretase components with anti-MAO-B IgG, is thus highly intriguing. This finding together with our observation that MAO-B expression in neurons is increased in AD, led us to perform detailed studies on the γ-secretase/MAO-B association in neuronal cultures and brain tissue. For studying protein–protein associations, PLA has some major advantages because this methodology is very sensitive and specific and can be performed on native proteins in their native cellular environment in situ. We used both the standard PLA setup and the GTB-PLA method, which utilizes a probe that is specific for the active site of γ-secretase [22, 25]. The GTB-PLA method verified that MAO-B can interact with mature γ-secretase. The association of γ-secretase with MAO-B in neurons was shown by PLA to be present in cultured primary neurons from mouse as well as in cortical and hippocampal neurons in mouse and human brain. To some extent, the association was also observed in astrocytes. Although AD brain appeared to give a higher degree of PLA signals for the γ-secretase/MAO-B association than control cases, the increase was not statistically significant due to the high standard deviation. It is likely, however, that the large variation between AD samples is at least partially caused by the combined effect of a loss of neurons and an increased γ-secretase/MAO-B association in the remaining neurons in AD.

MAO-B is considered to be localized in the outer mitochondrial membrane. However, this is not the sole subcellular location because it has also been found in other compartments, including ER and lysosomes [29]. γ-Secretase is believed to be localized predominantly to Golgi, ER, the plasma membrane, endosomes and lysosomes [30]. Thus, several potential compartments for the interaction can be envisioned. If mitochondrial MAO-B interacts with γ-secretase, it is likely that the interaction takes place in the ER–mitochondrial interface (MAM), because γ-secretase has been suggested to be enriched in the ER part of the MAM [31, 32]. Notably, it has been reported that MAO-A, an isoenzyme with different but overlapping substrate binding properties compared to MAO-B, interacts with PS1, and it was suggested that the interaction is mediated via the cytoplasmic loop/substrate binding site of PS1 [29]. MAO-B and MAO-A share approximately 70% sequence identity and have very similar chain folds [33], suggesting that MAO-A and MAO-B may have similar γ-secretase binding sites.

Interestingly, studies in cell lines have shown that MAO-B inhibitors can influence α-secretase activity, an effect that was suggested to be secondary to signaling events [34]. Our experiments indicate that MAO-B affects intraneuronal Aβ42 levels via a direct effect on γ-secretase. It will thus be interesting in future investigations to study whether proteolytic fragments derived from APP processing are involved in regulating such signaling events. In mouse brain, it was shown that not only the MAO-B inhibitors rasagiline and the propargylamine-based dual MAO-B/cholinesterase inhibitor ladostigil (TV3326) but also its enantiomer TV3279 (lacking MAO-B inhibitory activity) affect protein kinase C and APP levels in mice, indicating that a property other than the inhibitory activity of propargylamine may cause this effect [35].

Because there is a possibility that a γ-secretase-associated protein could affect Aβ production indirectly via altered activities of other secretases than γ-secretase or via alterations in subcellular trafficking, we performed an experiment in which both MAO-B and the immediate γ-secretase substrate, C99, were overexpressed. The finding that the combination of overexpression of MAO-B and C99 enhanced Aβ production strengthens the hypothesis that the γ-secretase/MAO-B association exerts an effect on γ-secretase activity. The higher effect of MAO-B overexpression at 50 MOI than at a dose of 100 MOI may be due to a compensatory or feedback effect. Alternatively, it could be due to overexpression causing defective subcellular sorting or aggregation of the overexpressed protein, as discussed previously [25].

The substantial γ-secretase/MAO-B association in neurons and low association in HEK293 cells [25], combined with the limited effect of siRNA silencing of MAO-B on Aβ secretion in HEK293 cells, triggered us to study whether siRNA silencing of MAO-B could have a more pronounced effect on Aβ production in neurons than in cell lines. Indeed this was the case, supporting the fact that the γ-secretase/MAO-B association is important for the effect of MAO-B on Aβ42 levels. In the initial experiments with fluorescently labeled siRNA, we observed that most of the neurons had taken up siRNA. At the lowest and intermediate concentrations of siRNA used for silencing of MAO-B, there was a significant reduction in intraneuronal MAO-B expression, whereas the effect of reduction of MAO-B was smaller at the highest siRNA concentration used. We observed fluorophore-conjugated siRNA in vesicular structures in transfection experiments at which higher siRNA concentrations were used (Additional file 1: Figure S5), as described previously [36]. It is possible that the siRNA/lipofectamine ratio at the highest concentration of siRNA may be too high to form the optimal complexes for sufficient cationic lipid-mediated transfection.

Our strategy developed here for relative quantification of intracellular Aβ42 levels on a single cell basis is a powerful method to investigate the effects of siRNA silencing, and was readily applicable to primary neuronal cultures. The data revealed that MAO-B silencing in primary cortex neurons significantly lowered the levels of intracellular Aβ42. Stunningly, the correlation between the intensity of Aβ42 and MAO-B staining even in the nontransfected cells supports the hypothesis that endogenous MAO-B regulates Aβ production in neurons. Notably, recent studies have shown that binding of the MAO-B ligand ^11^C-DED is increased at a very early stage in AD [16, 37, 38], demonstrating that both Aβ pathology and increased MAO-B expression are early events in AD. Further support for the connection between MAO-B and AD comes from clinical studies with MAO-B inhibitors, which have reported improved cognition and delayed AD progression [39, 40]. It appears that the effects of MAO-B inhibitors in clinical trials are not only due to the effect on catalytic activity, but could also be due to the lowered release of H_2_O_2_ and other reactive oxygen species, or hitherto nondefined functions [29]. Moreover, both the data presented here and previous reports showing enhanced MAO-B levels in PiB^+^ MCI cases, but not in PiB^–^ MCI cases [16], suggest that there is a correlation between MAO-B and Aβ pathology in AD. Thus, in light of previous reports and our findings, we suggest that the γ-secretase/MAO-B association and the effect on neuronal Aβ levels may be pathologically relevant and that the association may be a target for AD treatment.

## Conclusions

This study is a step toward understanding the early connection between MAO-B and AD pathogenesis. We show that neuronal MAO-B levels are increased in the frontal cortex and hippocampus in AD. Several protein–protein interaction assays supported the hypothesis that γ-secretase is associated with MAO-B in the neurons. Overexpression of MAO-B enhanced processing of the immediate substrate for γ-secretase (C99), resulting in increased Aβ formation. Moreover, there was a correlation between MAO-B and Aβ42 levels in cortical neurons, and silencing of MAO-B in these neurons reduced the intracellular levels of Aβ42. Thus, we conclude that MAO-B has a regulatory effect on intraneuronal Aβ levels, presumably mediated by γ-secretase. In addition, we envision the γ-secretase/MAO-B association as a potential target for pharmaceutical intervention of AD.

## Additional file

Additional file 1: Supplementary figures.
**Figure S1**. MAO-B antibody validation, precipitation and expression experiments. **Figure S2**. Western blot (WB) of postmortem AD and control human brain homogenate with MAO-B antibody. **Figure S3**. Validation of the Aβ42-specific antibody G2-11 by immunocytochemistry. **Figure S4**. MAO-B and Ab42 quantification in single cells from MAO-B silenced cortex neurons. **Figure S5**. Treatment of primary cortical neurons with fluorescently labeled siRNA. (PDF 1472 kb)

## Abbreviations

^11^C-DED: ^11^C-deuterium-l-deprenyl
AD: Alzheimer disease
Aph-1: Anterior pharynx defective 1
APP: Amyloid precursor protein
Aβ: Amyloid β-peptide
co-IP: Coimmunoprecipitation
CTCF: Corrected total cell fluorescence
CTF: C-terminal fragment
DIV: Days in vitro
ER: Endoplasmic reticulum
FAD: Familial Alzheimer disease
FRET: Fluorescence resonance energy transfer
GCB: γ-Secretase inhibitor with a cleavable biotin group
GFAP: Glial fibrillary acidic protein
GTB: γ-Secretase inhibitor with a transferable biotin group
HEK: Human embryonic kidney
HepG2: Hepatocellular carcinoma G2
HPLC: High-performance liquid chromatography
HTRF: Homogeneous time-resolved fluorescence
LC: Liquid chromatography
MAM: Mitochondria-associated membranes
MAO-B: Monoamine oxidase B
MOI: Multiplicity of infection
MS: Mass spectrometry
NGS: Normal goat serum
NICD: Notch intracellular domain
NMDAr: *N*-methyl-d-aspartate receptor
PBS: Phosphate-buffered saline
Pen-2: Presenilin-enhancer 2
PET: Positron emission tomography
PiB: Pittsburgh compound B
PLA: Proximity ligation assay
PS1: Presenilin 1
ROI: Region of interest
RT: Room temperature
WB: Western blot
