# Supplementary material for: Monoamine oxidase B is elevated in Alzheimer disease neurons, is associated with γ-secretase and regulates neuronal amyloid β-peptide levels
Source: Alzheimers Res Ther. 2017 Aug 1;9:57. doi: 10.1186/s13195-017-0279-1 (PMC5540560; doi:10.1186/s13195-017-0279-1)
Supplement: Additional file 1: Supplementary figures. — Figure S1. MAO-B antibody validation, precipitation and expression experiments. Figure S2. Western blot (WB) of postmortem AD and control human brain homogenate with MAO-B antibody. Figure S3. Validation of the Aβ42-specific antibody G2-11 by immunocytochemistry. Figure S4. MAO-B and Ab42 quantification in single cells from MAO-B silenced cortex neurons. Figure S5. Treatment of primary cortical neurons with fluorescently labeled siRNA. (PDF 1472 kb) [file 13195_2017_279_MOESM1_ESM.pdf]

Supplementary Figures
Alzheimer´s Research & Therapy

Monoamine oxidase B is elevated in Alzheimer disease neurons, is associated with γ-secretase and regulates neuronal amyloid β-peptide levels

Sophia Schedin-Weiss, Mitsuhiro Inoue, Lenka Hromadkova, Yasuhiro Teranishi, Natsuko Goto Yamamoto, Birgitta Wiehager, Nenad Bogdanovic, Bengt Winblad, Anna Sandebring-Matton, Susanne Frykman and Lars O. Tjernberg

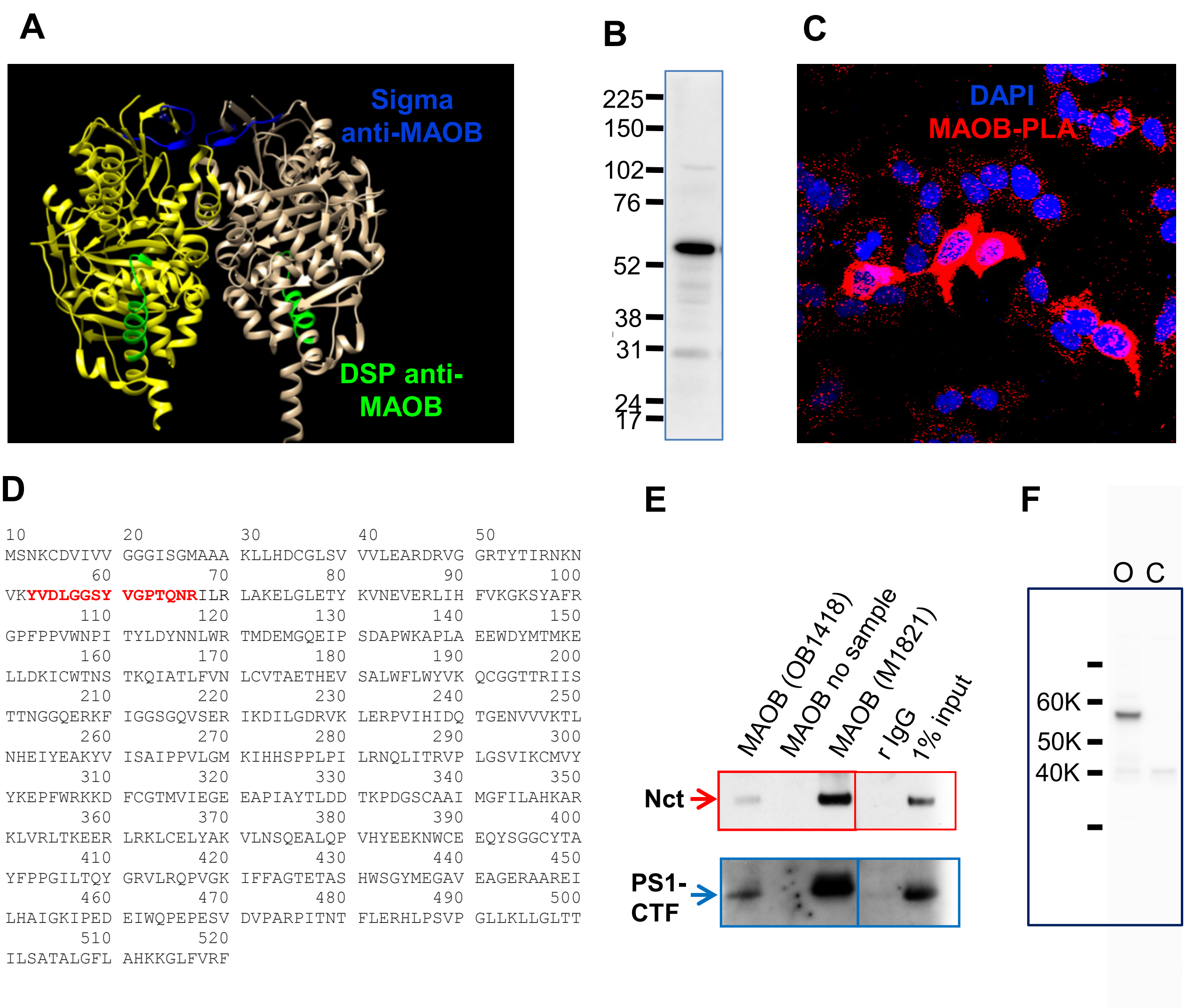

Figure S1. MAO-B antibody validation, precipitation and expression experiments.

a. Image of an MAO-B dimer drawn in Chimera (pdb structure 1S2Q). The epitopes for DSP anti-MAO-B IgG OB1418 (green) and Sigma Aldrich anti-MAO-B IgG M1821 (blue) are shown.
b. Validation of DSP anti-MAO-B IgG OB1418 by western blotting of human brain membranes isolated from frontal cortex shows one major band at the expected Molecular weight of ~ 58 kD.
c. Confocal image of HEK-APP cells overexpressing MAO-B and subjected to single-protein PLA to detect MAO-B (red), using DSP anti-MAO-B IgG OB1418. The overexpressing cells have considerably more PLA signals, as expected, which provides evidence that the antibody binds to MAO-B.
d. MAO-B amino acid sequence from rat, showing the sequence of the peptide (red) identified by Mass spectrometry of tryptic digests of rat brain synaptic membranes subjected to γ-secretase affinity purification method (with GCB) to identify γ-secretase associated proteins.
e. Co-immunoprecipitation of γ-secretase components with MAO-B. Microsomal membranes from human brain cortex were used.
f. Western blotting with anti-MAO-B (OB1418, 1:1000) of lysed HepG2 cells . O, MAO-B overexpressed cells; C, Control cells.

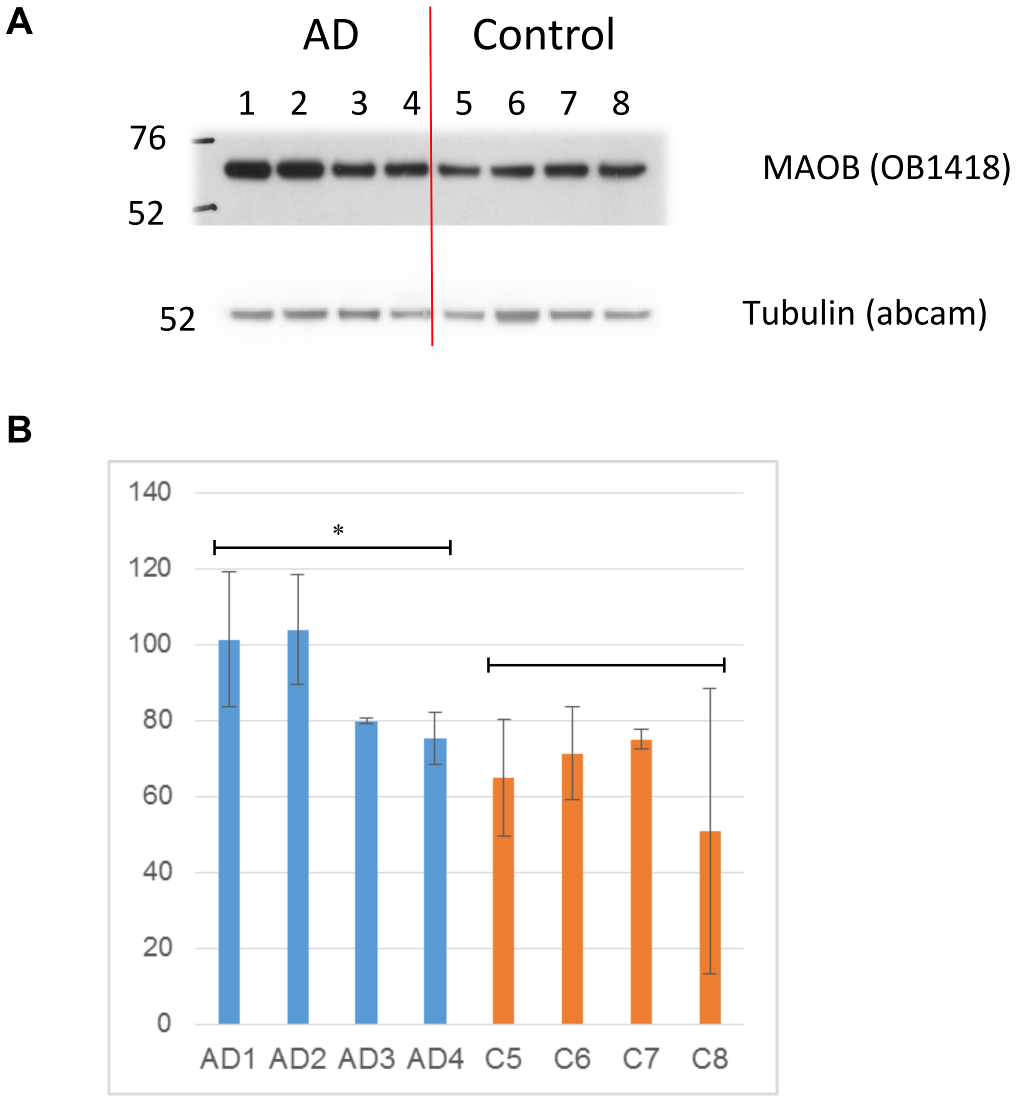

Figure S2. Western blot (WB) of postmortem AD and control human brain homogenate with MAO-B antibody.

a. SDS-PAGE and western blot was performed on homogenates from four AD and four control cases. Tubulin, detected as a control, showed no difference between the groups and was used as a reference protein.

b. Band intensities shown as mean values ± SD (quantified using Image J) from two different blots show a statistical difference between the AD and control group using t-test with two-tailed, two-sample unequal varince ($p=0.038$).

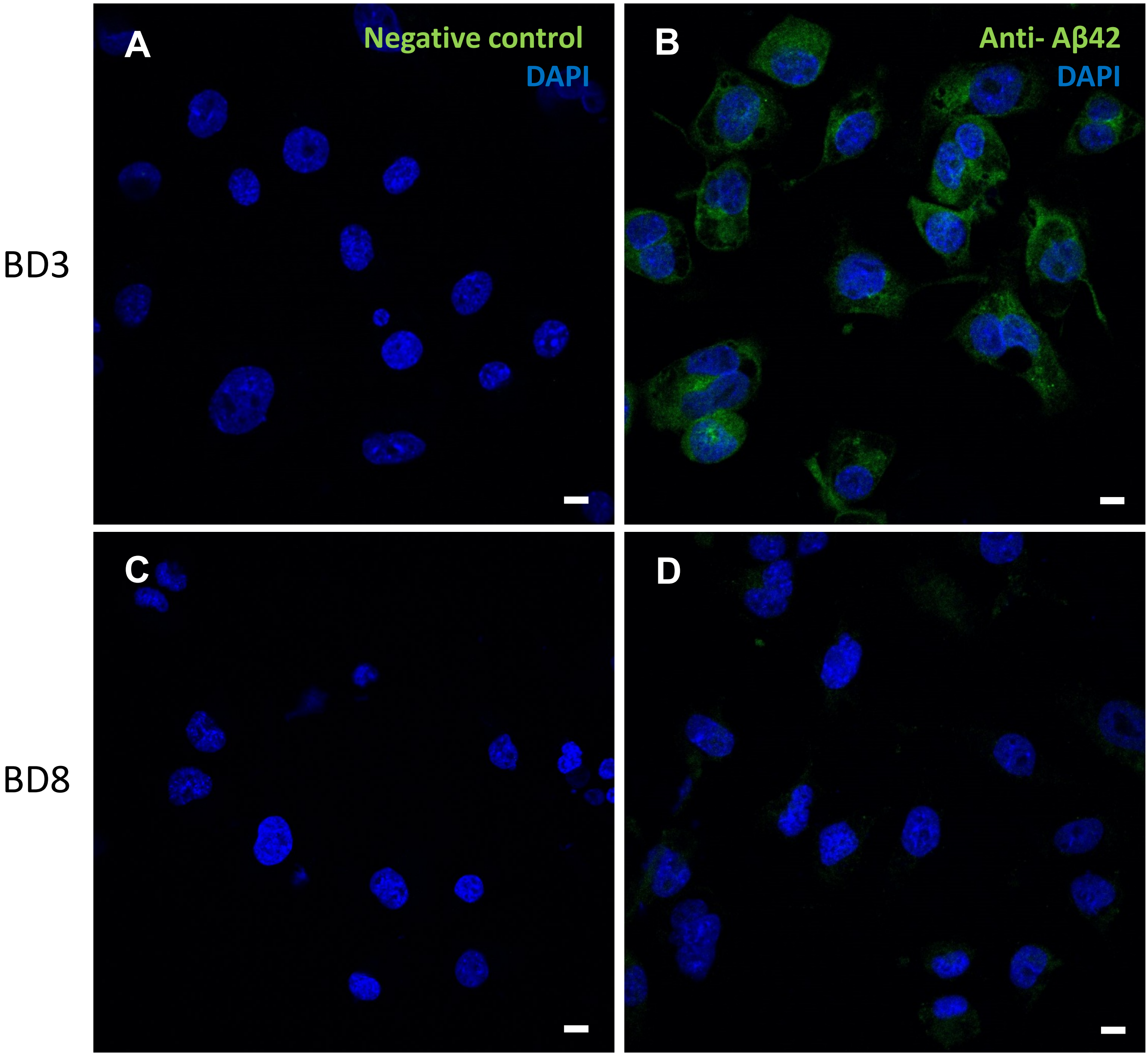

Figure S3. Validation of the Aβ42-specific antibody G2-11 by immunocytochemistry. BD3 (containing one PS1 allele) and BD8 cells (lacking both PS1 and PS2) were stained with the anti-Aβ42 specific antibody G2-11.

a. Negative control of BD3 cells (lacking the primary antibody).

b. Aβ42 staining of BD3 cells (with both primary and secondary antibodies).

c. Negative control of BD8 cells (lacking the primary antibody).

d. Aβ42 staining of BD8 cells (with both primary and secondary antibodies.

Nuclei were stained with DAPI (blue) and Aβ42 staining is shown in green.

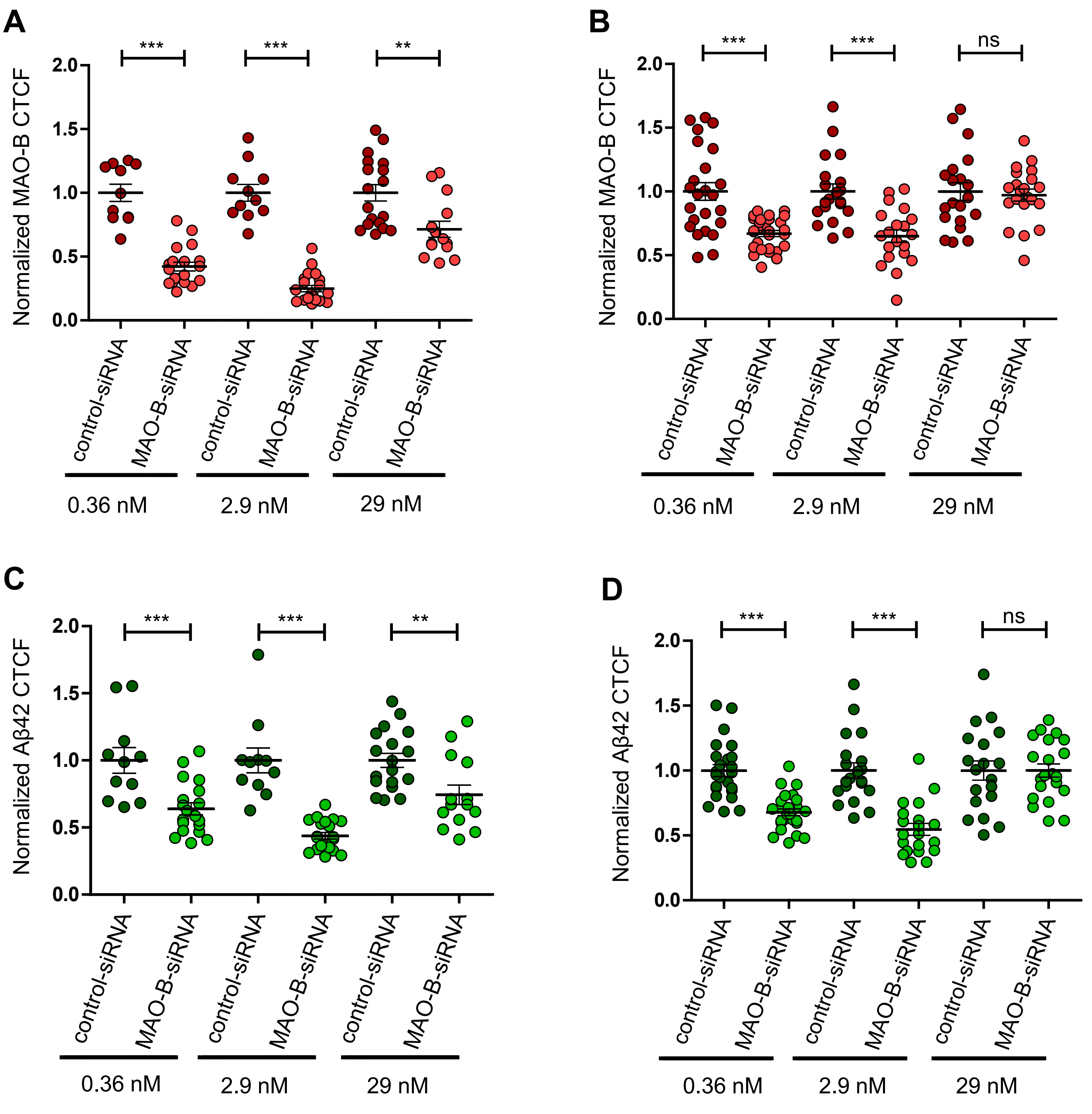

Figure S4. MAO-B and Aβ42 quantification in single cells from MAO-B silenced cortex neurons. Measurements were made from confocal images of MAO-B siRNA treated neurons (see also Fig. 8 in the main text). Here, data from individual cells is shown, where each circle represents data obtained from one cell.

a. MAO-B levels from the first transfection experiment.
b. MAO-B levels from the second transfection experiment.
c. Aβ42 levels from the first transfection experiment.
d. Aβ42 levels from the second transfection experiment.

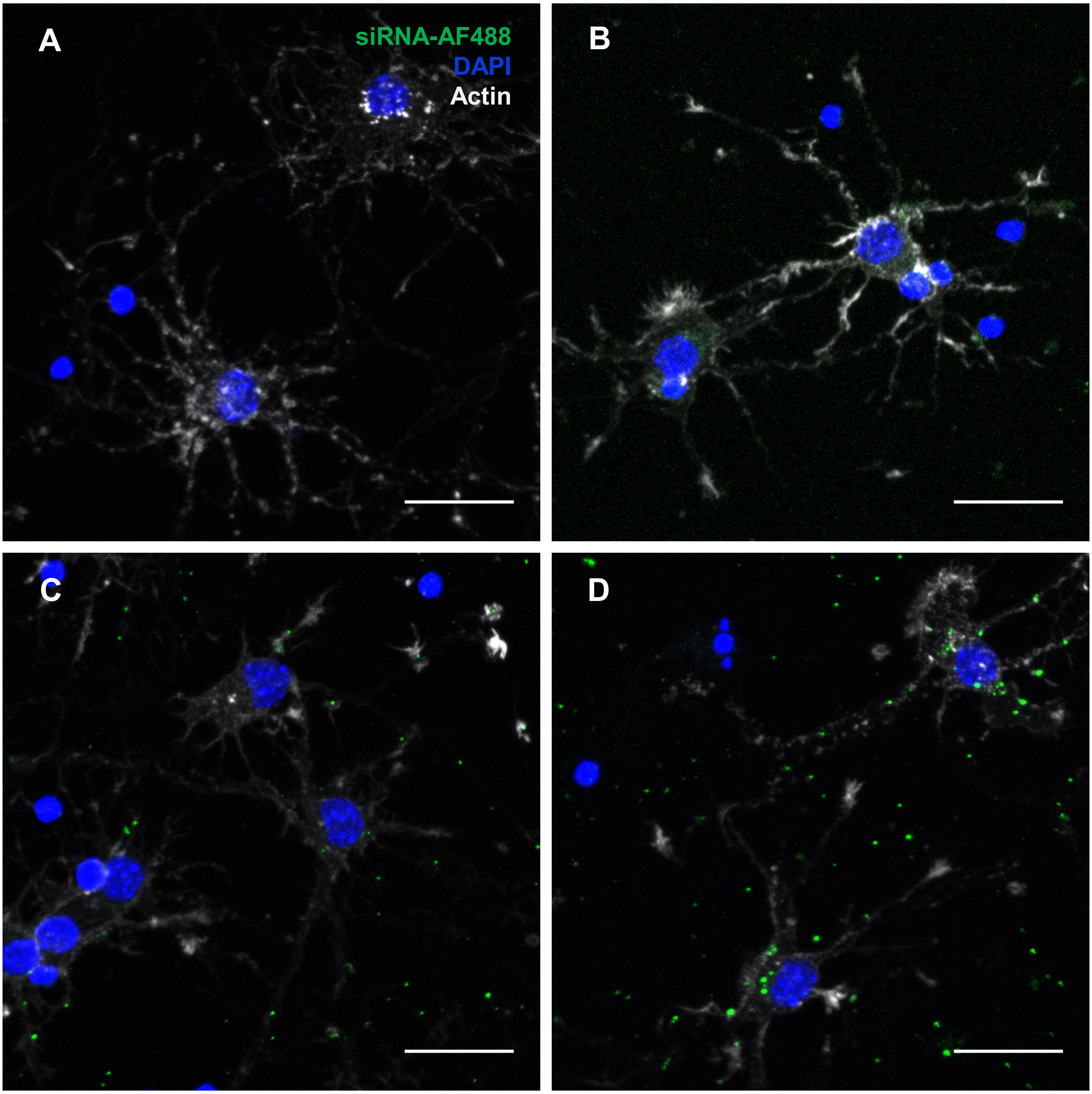

Figure S5. Treatment of primary cortical neurons with fluorescently labeled siRNA.
Confocal images of 7DIV mouse primary cortical neurons treated with AF488-conjugated siRNA (Qiagen, 1027280). Z-projections of maximum intensity images collected by Z-stack scanning at 0.54-µm intervals are shown, scale bar = 30 µm.

a. Neurons treated with Lipofectamine 3000 reagent only (no siRNA added).
b. Neurons treated with 0.36 nM AF488-conjugated siRNA.
c. Neurons treated with 2.9 nM AF488-conjugated siRNA.
d. Neurons treated with 29 nM AF488-conjugated siRNA.
